# Short-Term Effects of an AI-Based Wearable Adherence Monitor in Outpatient Psychiatry: Randomized Controlled Trial

**DOI:** 10.1192/j.eurpsy.2026.12055

**Published:** 2026-07-31

**Authors:** S. Yoon, W.-M. Bahk, S.-Y. Lee, B.-H. Yoon, E.-S. Lim

**Affiliations:** 1 Department of Psychiatry, Wonkwang University Hospital, Iksan-Si; 2 Department of Psychiatry, The Catholic University of Korea, Seoul, Korea, Seoul; 3 Department of Psychiatry, Naju National Hospital, Naju, Korea, Naju-Si; 4 Department of Psychiatry, Shinsegae Hyo Hospital, Gimje, Korea, Republic of

## Abstract

**Introduction:**

Medication nonadherence undermines treatment effectiveness in psychiatric care, yet objective, continuous measurement in routine practice is challenging. AI-enabled wearables may offer scalable monitoring but evidence from randomized evaluations remains limited.

**Objectives:**

the present study evaluated the effectiveness of an AI-based wearable and behavioral data algorithm for adherence monitoring in outpatient psychiatric patients, using a randomized design.

**Methods:**

We conducted a single-center, prospective, randomized controlled trial of an AI-based, wrist-worn adherence monitoring system in outpatient psychiatric care. Participants (age ≥12 years) were randomized 1:1 to smartwatch intervention or usual care for 4 weeks. Algorithm-derived adherence was calculated as the proportion of detected medication events relative to scheduled doses over prespecified 28-day baseline and post-intervention windows. The primary endpoint was change in adherence (Δ = post − pre). Analyses used complete cases with linear regression (adjusting for baseline adherence, age, sex, and medication covariates) and HC3 standard errors; ANCOVA served as a confirmatory model. Prespecified responder thresholds were Δ ≥10 and ≥20 percentage points (pp). Sensitivity analyses excluded benzodiazepine users.

**Results:**

Seventy-eight participants were analyzed (control n=41; intervention n=37; mean age 18.8±6.7 years). Baseline adherence was similar (control 22.4%±16.5; intervention 20.4%±17.1). Follow-up adherence increased modestly in controls (25.5%±15.2) and substantially in the intervention group (39.9%±34.5). The mean change favored intervention (+19.5%±38.1 vs +3.1%±11.5). In adjusted models, intervention assignment was associated with greater improvement (β=14.28, 95% CI 1.80–26.77; p=0.025); ANCOVA results were consistent. Responder analyses showed higher odds at the ≥20 pp threshold (45.9% vs 4.9%; OR=16.58, 95% CI 3.48–78.97; p<0.001), but not at ≥10 pp (54.1% vs 39.0%; OR=1.84; p=0.185). Excluding benzodiazepine users yielded similar effects.

**Conclusions:**

An AI-enabled smartwatch intervention improved short-term, algorithm-derived medication adherence compared with usual care in outpatient psychiatry. Larger, multi-center trials with longer follow-up and clinical endpoints are warranted.

**Disclosure of Interest:**

None Declared

